# The utilisation of primary health care system concepts positively impacts the assistance of patients with rare diseases despite limited knowledge and experience by health care professionals: A qualitative synopsis of the evidence including approximately 78 000 individuals

**DOI:** 10.7189/jogh.13.04030

**Published:** 2023-08-04

**Authors:** Raquel Lemos Ferreira, Israel Júnior Borges do Nascimento, Victor Izidro Alves de Almeida, Vanuza Regina Lommez de Oliveira, Larissa Gomes Marangne, Flávia dos Santos Gameleira, Tárcia Regina Coura Dutra, Daniela de Oliveira Santos, Marcelo Pellizzaro Dias Afonso, Paula Eduarda Alves dos Santos, Helian Nunes de Oliveira, Fernando Machado Vilhena Dias

**Affiliations:** 1 Faculdade de Medicina e Hospital das Clínicas, Universidade Federal de Minas Gerais, Belo Horizonte, Minas Gerais, Brasil; 2 Laboratório de Investigação de Pessoas com Doenças Raras (LIRA), Federal University of Minas Gerais, Belo Horizonte, Minas Gerais, Brazil; 3 Pathology and Laboratory Medicine, Medical College of Wisconsin, Milwaukee, Wisconsin, USA; 4 Division of Country Health Policies and Systems (CPS), World Health Organization Regional Office for Europe, Copenhagen, Denmark; 5 Programa de Pós Graduação em Saúde Pública, da Faculdade de Medicina, Federal University of Minas Gerais, Minas Gerais, Brazil; 6 Programa de Pós-Graduação em Medicamentos e Assistência Farmacêutica, Faculdade de Farmácia, UFMG, Minas Gerais, Brazil; 7 Programa de Promoção de Saúde e Prevenção da Violência, Departamento de Medicina Preventiva e Social, Faculdade de Medicina, UFMG, Minas Gerais, Brazil

## Abstract

**Background:**

Individuals with rare diseases (RD) have been historically understudied. Previous publications reported that existing primary health care (PHC) workforces and associated infrastructure had been shown to improve their access and health-related outcomes in low- and middle-income countries (LMICs). As current evidence about the impact of PHC on patients diagnosed with RD is yet highly dispersed, this scoping review aimed to collate available evidence of the impact of PHC on patients with RD and summarize published information from multiple stakeholders about the perceived usefulness and barriers to effective use of the PHC system.

**Methods:**

We searched Embase, Health System Evidence, PubMed, LILACS / BVS, and The Cochrane Library, from inception to September 1, 2022, for publications providing clear expert- or experience-based insights or data from patients living with RD at the PHC level of care. We included publications highlighting barriers to integrated care of patients with RD, reported by multiple social actors involved in caring for patients with RD. Two investigators screened publications, extracted data, and clustered information among records deemed eligible for inclusion. Data synthesis was performed using narrative and thematic-based analysis. Major findings identified and coded through a semantic-driven analysis were processed in vosViewer software and reported using descriptive statistics.

**Findings:**

Eighty publications were included in this review. Quali-quantitative analyses evidenced that the PHC level is essential for approaching patients with RD, mainly due to its longitudinal, multidisciplinary, and coordinated care delivery. In addition, several publications highlighted that the medical curriculum is inappropriate for preparing health care providers to deal with patients presenting unusual signs and symptoms and being diagnosed with RD. PHC teams are essential in orienting patients and families on emergency events. Technology-related concepts were reported in 19 publications, emphasizing their effectiveness on early diagnosis, optimal treatment definition, improvement of quality of life, and long-lasting follow-up.

**Conclusions:**

We provided valuable information on the effectiveness of the PHC in fostering a creative, integrative, and supportive environment for patients living with RD. Our results can be helpful to several stakeholders in deciding what actions are still pending to achieve a solid and positive experience for patients with RD in the PHC.

**Registration:**

PROSPERO (CRD42022332347)

According to the World Health Organization (WHO), rare diseases are pathologies or medical conditions affecting no more than 1.3 in 2000 individuals [1]. The interests of several stakeholders (eg, health care professionals and medical organizations) in understanding and evaluating these diseases have significantly increased over the last years as it is estimated that more than 5500 have been reported worldwide, affecting more than 300 million people [2]. Despite the global attempt to obtain concrete epidemiological data regarding rare diseases, health-related agencies have been working to improve the human condition and achieve equal dignity for every citizen. For instance, based on a multi-stakeholder alliance between the public and the private sector, the United Nations and its agencies created the Agenda for Sustainable Development, including 17 assessable Sustainable Development Goals [3,4]. This initiative not only fights against poverty (commonly observed among patients with a rare disease) but also aims for the delivery of high-quality education, achievement of gender quality, reduction of country inequalities, and revitalize the global partnership for sustainable development [4].

Along with several available pharmacological and non-pharmacological interventions to treat and long-term manage rare diseases, the primary health care systems and its professionals also stand as a fundamental level of care due to three core elements (meet patients' health needs throughout their life, address the broader determinants of health through multi-sectoral policy and action, and empower individuals, families, and communities to take charge of their health) [5]. In countries where a primary health care system is functional and adequately implemented, caring for patients with rare diseases is directly linked to primary health care and the professionals practicing at this level of care. However, many low-, middle-, and high-income countries do not still guarantee the humanization of care, coordination and maintenance of care, implementation of practices that enable early diagnosis, and deployment of actions that expand the autonomy of users and their caregivers [6].

Currently, hundreds of publications evaluating multiple features of rare diseases have been registered, suggesting the need for structured and integrated multidisciplinary care and the evolvement of frequently observed health care systems worldwide. Even though these publications reveal crucial elements regarding the diagnosis, treatment, and follow-up of patients presenting rare diseases, no summarization of these components have been published and analysed. Therefore, this systematic review aimed to answer the following guiding questions: “1. What is the state-of-the-art related to the role of the primary healthcare system in the care of individuals affected by rare diseases?”; “2. What is the current evidence suggesting the effectiveness of primary healthcare system approach on managing patients with rare diseases?”; and “3. What are the practice and structural gaps in the care network that hinders the integrality of care to patients with rare diseases?”.

## METHODS

To map relevant publications in rare diseases, this study followed the published methodological framework for conducting a scoping review developed by Arksey and O'Malley [7]. Our starting point review questions were “What topics and information have been evidenced and published in the body of the literature regarding the use of primary healthcare systems for managing cases of rare diseases?”, “What is the role of primary healthcare assistance in terms of rare diseases patient's care flow”, and “What barriers and facilitators currently exist for the adequate establishment of primary healthcare system applied to rare diseases worldwide?”. Furthermore, our findings were reported under the Preferred Reporting Items for Systematic Reviews and Meta-Analysis extension for Scoping Reviews (PRISMA-ScR) [8]. Before executing this review, we registered our protocol on PROSPERO (CRD42022332347).

Regardless of study design and publication type, relevant records were obtained from five databases (Embase, Health System Evidence, PubMed, LILACS / BVS, and The Cochrane Library). The searches were performed on September 1, 2022; therefore, we included records from inception until the before-mentioned date. Publications were included if they: 1. directly reported and evaluated the role and effect of primary health care-derived interventions to the genetic- or clinical-based diagnosis, treatment, management, and prevention of late diagnosis of rare diseases; 2. provided legal or institutional information regarding rare diseases patient's rights; 3. compared the impact of primary health care-related activities to secondary or tertiary level of care; and 4. reported barriers, facilitators, and opportunities to implement long-lasting direct health care programs successfully. It is worthwhile mentioning that the acronym PICO (Population, Intervention, Comparator, Outcome) was used in this review to guide the entire review process. As far as “population” is concerned, patients diagnosed with rare diseases, as internationally defined in the literature, were considered eligible for inclusion. Furthermore, the primary health care system (defined within each included study) was regarded as the direct “intervention” evaluated throughout retrieved studies. We did not exclude studies based on the existence or absence of a comparator or a particular discussed outcome to increase the potential number of records deemed to be included.

We did not impose restrictions on published language and conference proceedings were considered eligible for inclusion. However, records were excluded if they did not assess the impact of primary health care on rare diseases. We used the Orphanet database for rare diseases and orphan drugs, created by the French National Institute for Health and Medical Records [9]. Following duplicate removal (using EndNote 20), obtained records were uploaded into Covidence, primarily for the title and abstract screening phase and subsequentially to the full-text screening stage. Screening processes were performed independently by two review authors. Any decision disparity was resolved by group discussion.

An information specialist and medical experts in rare diseases and public health care collaborated to define each database's most sensitive search strategy. Search string levels associated with primary health care included “Primary Health Care”, “Physicians, Primary Care”, and “Primary Care Nursing” and their synonymous. Additionally, rare disease elements were mostly represented by key terms such as “Rare Diseases” and “Orphan Diseases”. A complete description of keywords and identifiers used in each database is displayed in Section 1 in the **Online Supplementary Document**. References list of shortlisted records for full-text analysis was evaluated to ensure an exhaustive search.

Data extraction of studies deemed eligible was also performed independently by two research authors and the following variables were extracted: 1. study identification; 2. publication year; 3. journal name; 4. SCImago Journal Rank (SJR) indicator for each journal; 5. study design; 6. publication type; 7. study objective; 8. number of patients or individuals considered in the publication; 9. targeted health care professionals; 10. rare disease evaluated; 11. orphanet code; 12. country; 13. summary of findings or discussed topic; 14. any information platform provided throughout the record; 15. barriers to case management; 16. facilitators for case management; and 17. any future opportunities raised by original authors. If any data extraction conflict occurred, a discussion between the two conflicting authors was carried out to understand which elements should be finally displayed in the final materials.

As we retrieved and selected qualitative and quantitative findings, we created a thematic synthesis of the results. The initial main findings of included studies were coded and thematically collated using NVivo software. Statistics data from quantitative studies were shown through text or table-based methods, while qualitative data were clustered by similarity and subsequently graphically presented using the vosViewer software. Moreover, a general summary of findings was created to systematically abstract the review's findings.

We primarily planned to analyse the risk of bias assessment for those included records in which the evaluation was technically feasible and indicated. However, as our study focused on exploring the status quo of the available evidence regarding the delivery of care for rare diseases at the primary level of care, we decided not to evaluate it. Moreover, the quality of the evidence was not appraised because of the methodological and technical singularities of scoping reviews.

## RESULTS

**Figure 1:**
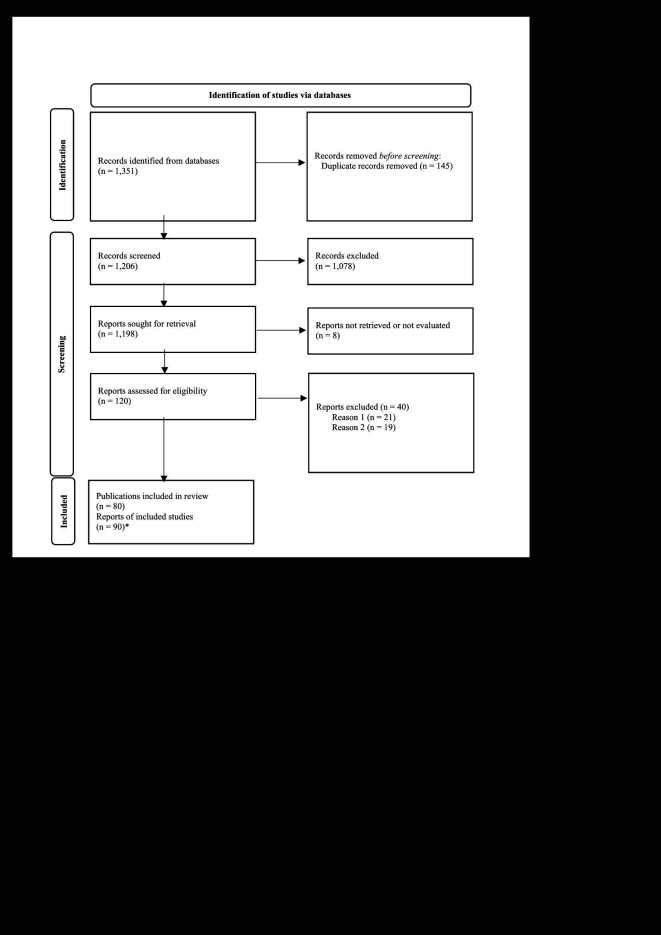
PRISMA flowchart diagram. *Studies corresponded to literal translation of main records, therefore, counted only once.

Our search strategy retrieved 1351 records (as 1341 single studies), of which 145 were duplicates. The remaining 1206 records were screened, and 1078 studies were judged irrelevant. From the 128 shortlisted records, 90 were deemed eligible for inclusion. However, as some included records were literal translations of each other, we ended up with 80 unique publications in the qualitative summarization [10-90]. Justifications for excluding records during full-text screening are available in Section 2 in the **Online Supplementary Document**. A flow diagram of article selection is shown in **Figure 1**. Eight records were classified as “awaiting classification” either because the full text was not available or the translation of the study could not be done by the time of submission [38,91-98]. Generically, most included studies were published in the Atencíon Primaria (n = 9) [13,25,33,40,41,43,65,89,91], Orphanet Journal of Rare Diseases (n = 5) [20,28,38,73,83], the Journal of the Sociedad Española de Médicos de Atención Primaria (n = 4) [10,14,58,75], and the Revista Brasileira de Medicina da Família e Comunidade (n = 3) [11,53,76]. Included records were predominantly cross-sectional studies (n = 26), narrative papers (n = 18), and reviews or case reports (n = 10). Numerically, as far as studies that evaluated patients and their rare diseases are concerned, 73 722 individuals were grouped, while approximately 4445 health care providers were included throughout the eligible studies. Most studies evaluated physicians' knowledge, understandability, perception, and professional attitudes (predominantly general practitioners) and nurses. In addition, included studies assessed common experiences lived by patients and their caregivers or described the importance of establishing guidelines for caring for patients with rare diseases at the primary care level.

The main characteristics of the included citations are summarized in **Table 1** and **Table 2**. Among the 80 included publications from 2004 (n = 1) [69] to 2022 (n = 8) [17,20,33,34,37,42,56,85], there were publications from approximately 40 countries (mostly from the European Region and Latin America). Although not frequently described or discussed within included publications, several platforms are utilized by health care professionals in the event of case suspicion, including Orphanet, Sistema de Información de Enfermamidades Raras en Español (SIERRE), Online Mendelian Inheritance in Man (OMIM), and Phenomizer. The included publications evaluated 64 different rare diseases or conditions, including highly limiting and severe diseases (eg, inborn errors of metabolism, amyotrophic lateral sclerosis, and primary immunodeficiencies). Conditions that appeared more than one time among included publications were cystic fibrosis (n = 3) [55,74,90], inborn errors of metabolism [15,43], amyotrophic lateral sclerosis [25,32], and autoimmune hepatitis [50,84] (n = 2).

**Table 1:** Main characteristics of included studies

Study ID	Journal	SD	Objective or major approach	No of patients or individuals	Aimed health care professionals
Acevedo Gragera, 2006*	SEMERGEN	CS	To report ten years of follow-up in PC of two patients diagnosed with Werner Syndrome and to describe the most recent theories of its aetiology	2	N / A
Amaral, 2016*	Rev Bras Med Fam Comunidade	CR	To report a rare case of McArdle Disease	1	N / A
Anon, 2006	Atención Primaria	-	-	-	-
Avellaneda 2007‡	Anales del Sist Sanit de Navarra	R	To highlight that rare diseases appear like a universe that requires a new socio-sanitary approach from the health system	N / A	N / A
Avellaneda Fernandez, 2006*	Atención Primaria	NM	To define the relevance of the problem of the need for PC training in rare diseases and to identify the need for RD training in PC	22 HP	Phy, nurses, and SHC
Avellaneda Fernandez, 2012*	SEMERGEN	CSS	To identify the elements that lead to the perception that GPs have as regard RD as a whole, and to analyse the characteristics of these patients with RD	260 Phy	Phy
Beck, 2020*	Transl Sci Rare Dis	R	To provide PCP with information about the medical and psychosocial issues commonly experienced by patients with inborn errors of metabolism and their families to allow for ongoing support outside of the genetics clinics	N / A	HP in general
Bedin, 2021*	Rev de Aten Prim a Saúde	NM	To reflect on rare diseases approach in the context of PC	1 Pt	Medical students
Benson, 2022*	European Journal of Neurology	R	To describe the holistic patient experience from pre-diagnosis through to long-term treatment	15 Pt	N / A
Boffin, 2018*	Intl J of Environ Res and Public Heal	CSS	To examine care characteristics of care diseases patients and analyse the importance of RD in GP by its caseload	111 SGP + 121 Pt	Phy
Brasil, 2014§	Ministério da Saúde Brasileiro	GM	To establish guidelines for the care of patient with RD in the primary health care system	N / A	N/A
Buendia, 2022*	Orphanet Journal of Rare Diseases	CSS	To assess the feasibility of applying MendelScan with 76 RD algorithms, in a PC environment in the lower lea valley	68 705 Pt	N/A
Bueno, 2015*	Soc Iberoameric de Inform Científica	CSS	To assess the degree of knowledge on rare diseases held by PCP	128 Phy	Phy
Buenos Aires, 2013§	La APS renovada en la Provincia de Buenos Aires	GM	It establishes the creation of the Center Provincial Reference, Monitoring and Dissemination of RD and establishes its bases concepts in this document, constituting its pillars of work	N / A	N / A
Byrne, 2020*	Irish Journal of Medical Science	CSS	To complete a pilot survey to estimate the general practice clinical workload attributable to selected RD and assess the use of relevant information sources	31 Phy +171 Pt	Phy
CAN Org for Rare Diseases, 2015§	N/A	GM	To highlight strategies for care of RD	N / A	N / A
Carroll, 2021*	Canadian Family Physician	CSS	To explore PCP preferred roles and confidence in caring for infants receiving a positive cystic fibrosis newborn screening result, as well as management of cystic fibrosis family planning issues	321 HP	PCP and specialists
Castro-Rodríguez, 2021*	Atención Primaria	CSS	To know the incidence and prevalence of amyotrophic lateral sclerosis in a PC management area, the clinical characteristics and the use of health resources	81 Pt	N / A
Crit Care Services Ontario, 2017§	N/A	GM	To report data RD from the development of a provincial framework to maximize value from the system of care for patients with R in Ontario	N / A	N / A
Derayeh, 2018*	Intract and Rare Diseases Research	R	To systematically review the literature on the RD information system to identify architecture of this system from a data perspective	N / A	N / A
Domínguez Enríquez, 2013*	Revista Médica VozAndes	CR	To present a care report of a RD and the delivery of care in the primary care sector	1 Pt	N / A
Druschke, 2021*	Orphanet Journal of Rare Diseases	CSS	To understand the knowledge about a centre for RD and how it works, in case of cooperation, satisfaction with the services provided by centres, and expectations and needs they have with regard to the centres	263 Phy	Phy
Dudding-Byth, 2015*	Australian Family Physician	R	To outline the challenges faced by the RD community, and the role of the PCP to advocate for answers as their patients transition through the health care system	N / A	GPs
Ehsani-Moghaddam, 2018*	PLoS ONE	CSS	To evaluate the NBC algorithm as a simple method of identifying a “group” of patients with the highest likelihood of having MPS II as a rare disease with relatively common features using a “real” data set	N / A	N / A
Elliott, 2015*	Australian Family Physician	R	To review the impact of rare diseases on families and health services, and the role of the GP and policy response in Australia	N / A	GPs
Esteban-Bueno, 2022*	Atencion Primaria	PM	The follow-up of patients with Wolfram syndrome can be used to design a protocol to support these patients, with the participation of researchers and health care professionals specialized in the disease, the patients themselves and their familial environment	N / A	HP in general
Esteban-Bueno, 2016*	Rev Clín de Med de Fam	CSM	The objective of this case study was to analyse the importance of the role of the PCD in the presumptive diagnosis, referral, monitoring and support of patients with ALS and their families	2 Pt	GPs
Evans, 2016‡	British Journal of General Practice	NM	To identify important points to answer, how recognize the rare disease in primary care	N / A	GPs
Evans, 2022*	European Journal of Human Genetics	IRS	To evaluated the implemented MendelScan, a primary care rare disease case finding tool, into a UK NHS population	68 705 Pt	N / A
Fabrizzio, 2018*	Rev da Escola de Enfermagem da USP	CR	To describe the care management of an individual patient affected by Devic’s Disease in the PHC context	1 Pt	N / A
Falah, 2022*	BMC Primary Care	CSS	To evaluate quality, satisfaction, and barriers in genetics education in residency training programs	59 Phy	Phy
Francisco, 2018‖	Orphanet Journal of Rare Diseases	NM	To optimize the available resources and improve care quality, integrating patient care levels and circuits that guarantee access to clinical expertise unit networks (XUEC)	N / A	GPs
Gammie, 2015*	PLoS One	R	To review existing regulations and policies utilised by countries to enable patient access to orphan drugs	35 countries	N / A
García-Ribes, 2006‡	Atencion Primaria	NM	To present the sem FYC Working Group on Genetic and Rare Diseases and its objectives	N / A	GPs
García-Ribes, 2013*	Revista Clíc de Med de Familia	NM	To present an online tool that has been developed to manage rare diseases from the primary health care centre to facilitate the family doctor’s task of caring for these patients: the DICE-APER protocol	N / A	HP
García-Ribes, 2013‡	Atencion Primaria	NM	To present initiatives on the role of primary care in rare diseases	N / A	N / A
Gimenez-Lozano, 2022*	Intl J of Environ Res and Public Health	CSS	To understand the needs of patients and families seen in primary care with a confirmed or suspected diagnosis of rare disease and better grasp the impact the disease has on their lives	163 Pt	N / A
González-Lamuño, 2009*	Atención Primaria	R	To present the situations when a primary care physician should suspect a rare metabolic disease	N / A	N / A
Gupta, 2011¶	Chest	NM	To explore several strategies that may improve researchers’ ability to identify and recruit research participants with rare lung diseases and to provide an overview of strategies based on available evidence, previously used approaches, and reasoning	N / A	N / A
Hariyan, 2020*	PLoS ONE	CSS	To evaluate the knowledge about primary immunodeficiencies among physicians before and after the implementation of an educational program	149 Phy	Phy
Hayward, 2017‡	British Journal of General Practice	CR	To analyse how will genomics impact on primary care and what is needed for primary care to be genomics-ready	N / A	N / A
Iskrov, 2019*	Annali dell'Istituto Superiore di Sanita	NM	To analyse the five identified axes of public health challenges in the 2017 State of Health in the EU report to start a broad debate on the issue of sustainability of health care systems for rare diseases	N / A	N / A
Jo, 2019*	BMJ Open	CSS	To explore characteristics of visits for patients with rare diseases seen by primary care physicians (PCPs)	1508 Phy	PCP
Kim, 2015*	BMC Gastroenterology	CS	To investigate if having access to primary care or insurance prior to diagnosis is associated with better outcomes for patients in an urban, public hospital with mostly socioeconomically disadvantaged Hispanic patients	150 Pt	N / A
Knight, 2006*	Medical Journal of Australia	HM	To develop a comprehensive approach to the management of chronic disease in primary care	N / A	CMC
Koch, 2012‖	Molecular Syndromology	GM	To introduce the DORA project, a joint effort between the Children’s Hospital (University of Sao Paulo Medical School) and the Sao Paulo Secretary of State for Health, in order to organize early diagnosis and integrated care of congenital malformations and rare diseases in the State of Sao Paulo	N / A	Phy
Loio, 2017*	Rev Bras Med Fam Comunidade	CR	To demonstrate the importance of early recognition of rare disease characteristics in family and community health for a good prognosis	1 Pt +1 Phy	GPs
Lopes, 2018*	Clinics (Sao Paulo)	GM	To present a survey of vulnerabilities and to suggest approaches for the treatment of rare diseases according to the perceptions of a group of affected individuals, patient association representatives and health care professionals	27 Pt	HP in general
Luz, 2015*	Acta Paulista Enfermagem	NM	To characterize the diagnostic and therapeutic journey of families of people with rare diseases within the network of Brazilian public services	N / A	Nurse
Maggi, 2022*	Family Practice	CS	To investigate the clinical correlates of SMA among primary care patients.	800 Phy	GPs
Mainous, 2018*	JABFM	CSS	To evaluate the effectiveness of a clinical decision support (CDS) – based intervention system for transfusional iron overload in adults with SCD to improve management in primary care	71 Pt	GPs
Martínez-Sabater, 2012*	SEMERGEN	CR	To report how rare diseases, by their epidemiological characteristics, and sometimes by the non-specific symptoms, are difficult to diagnose routinely at the Primary Care Level	N / A	Nurse
McClain, 2014*	Clinical Pediatrics	GM	To assess primary care paediatric providers’ comfort with co-managing patients with rare conditions	108 Phy	Paediatricians
Mehta, 2017*	Mol Genet Metab	GM	To explore the patient journey to diagnosis of Gaucher Disease from the perspectives of Gaucher expert physicians and patients	1595 Pt	Genetic therapies
Melo, 2015*	Journal of Community Genetics	GM	To analyse genetic competencies of primary health care professionals in Brazil	21 Phy 21 + Nurses 16 + 8 Dentists	CHPEG
Melo, 2017*	Interface	GM	To provide a theoretical reference to support the outline of programs of education and training in Health, contributing to including Genetics in the SUS	N / A	Geneticists
Menon, 2015*	Healthcare Policy	R	To compare current mechanisms across provinces and territories, and explore their impact on access	N / A	N / A
Morales-Piga, 2013*	Atencion Primaria	NM	To evaluate the population of patients with FOP in Spain	24 Pt	N / A
Murphy, 2021*	Innovations in Pharmacy	CSM	To show the need for additional treatment guidelines for pain in patients with Budd Chiari Syndrome	1 Pt	N / A
Ministério da Saúde Brasileiro, 2015**	Brazilian Government	GM	To establish the National Policy for Comprehensive Care for People with Rare Diseases, approves the Guidelines for Comprehensive Care for People with Rare Diseases within the scope of the Health System (SUS) in Brazil and institutes financial cost incentives	N / A	N / A
Ortega Calvo, 2004‡	Cuadernos de Gestión para el Profesional de Atención Primaria	NM	To answer the question: “Are rare diseases a scientific paradigm in primary care?”	N / A	N / A
Ortega Calvo, 2007*	Anales de Medicina Interna	CSS	To answer the following question: How can we classify the findings in rare diseases from primary care in such a way that the information is as profitable as possible for the scientific community?	N / A	N / A
Ortega Calvo, 2012*	Atencion Primaria	NM	To present a series of diagnostic conceptual maps that help GPs and paediatricians make decisions about patients hypothetically affected by rare diseases	N / A	GPs and paediatrician
Pearce, 2018‡	Rheumatology	NM	Development of ‘effective IT support’ for rare conditions presenting with common symptoms, specifically advocating use of computer prompts to alert primary care physicians to consider a rare disease diagnosis	N / A	GPs
Pericleous, 2018‖	United European Gastroenterol J	CSS	To create a real-world registry linking primary and secondary care for PBC	375 Pt	Specialists
Ramalle-Gómara, 2020*	Orphanet Journal of Rare Diseases	CSS	To report the training needs and the perceived shortcomings of Spanish physicians of the public health system in the diagnosis, treatment and monitoring of patients with rare diseases	132 GPs and 37 specialists	GPs and specialists
Reimann, 2007*	BGG	CSS	To obtain insight into preferred medical care concepts and preferences in the way that care is provided	German patient-organisations	GPs and specialists
Rodríguez de Mingo, 2014*	SEMERGEN	CSM	To report the case of a 40-y-old Caucasian woman who came to the clinic with these symptoms and was diagnosed with Takayasu's arteritis	1 CR	PHP
Santos, 2020*	Rev Bras Med Fam Comunidade	NM	To collaborate with the development of methods for the recognition of individuals with or at risk of developing genetic diseases in the primary health care	1160 Pt	PHP
Senior, 2008¶	The Lancet	NM	General comment on rare diseases and primary health care	N / A	N / A
Siderius, 2012‖	Arch Dis Child	CSS	To establishes opportunities for PHC to detect children with rare and chronic conditions and provide PHC with tools for personalized prevention for children	931 newborns	PHP
Silva, 2020*	BGG	CSS	To understand the challenges faced by family caregivers of children and adolescents with Epidermolysis Bullosa in the search for assistance in the Health Care network	11 individuals	N / A
Stroes, 2017*	Atherosclerosis Supplements	NM	To define a diagnostic algorithm for FCS	N / A	HCP in general
Terry, 2010‡	JAAPA	NM	It discusses the intersection between PC and genetic diseases	N / A	PHC
Timmer, 2021*	Haemophilia	CSS	To explore experiences of stakeholders with primary care physiotherapy for patients with haemophilia and develop recommendations to optimize physiotherapy care coordination	Physioterapists	Physiotherapists
Vandeborne, 2019*	Orphanet Journal of Rare Diseases	CSS	To investigate how information and education could be tailored to the needs and preferences of physicians in Belgium to increase their rare disease awareness and support them in diagnosing patients with a RD	Phy	Phy
Vieira, 2009*	RAMB	CSS	To analyse the qualitative coverage of therapeutic policies in the Health System in Brazil, at the federal level, for diseases mentioned in lawsuits	27 Pt	HPs
Warnick, 2022*	Genetics in Medicine	CSS	To investigate the satisfaction and improvement demand of quality genetics education in residency training programs and thus provide a basis for its development and advancement	59 Phy	Phy
Wray, 2021*	Int J Pediatr Otorhinolaryngol	CSS	To identify the information and support needs of HPs in primary and secondary care looking after a child with LSCTS, the views of those providing education to these children, and elicit parents’ perceptions about community-based services, to improve overall care for children and families	90 HP + 18 professors	N / A
Yeung, 2016*	Haemophilia	R	To summarize the evidence from reviews for the effects of integrated multidisciplinary care for chronic conditions in adults and to provide an example of using this evidence to make recommendations for haemophilia care	N / A	N / A
Zack, 2006*	Community Genetics	CSS	To determine whether a knowledge gap is recognised, how GPs currently at- tempt to overcome it, and what features of an information resource are preferred by GPs	37 Pt	N / A

CME – consultant medical educator, CS – case series, CR – case report, CSS – cross-sectional study, NM – narrative model, HM – historical model, GM – government material, PHP -primary health care professionals, ALS – amyotrophic lateral sclerosis, UK-NHS – United Kingdom, National Health System, FYC – “proper name”, SMA – spinal muscular atrophy, PBC – primary biliary cholangitis, HPs – health care professionals, Phy – physicians, PC – primary care, PCD – primary care doctor, PCP – primary care physicians, PCW – primary care worker, PHC – primary health care, HCCC – haemophilia comprehensive care centre, BGG – Bundesgesundheitsblatt-Gesundheitsforschung-Gesundheitsschutz, SD – study design, N / A – no answer, R – review, RD – rare disease, RAMB - Revista da Associação Médica Brasileira, JAAPA - Journal of the American Academy of Pa, Pt – patient, SHW – social health workers, LSCTS - long segment congenital tracheal stenosis

*Journal Article.

‡Editorial.

§Government or organization publication.

||Conference abstract.

¶Comment.

**Law.

**Table 2:** Clustered findings occurrence among identified records

**Finding 20**	Current state of medical education for approaching rare diseases is inadequate	[5,8,10,13-15,17-19,22,23,25,28,31,34,35,45,49,54,56-59,62,63,66,73-76,78,79,81]
**Finding 12**	Longitudinal care for patients with rare diseases is fundamental and should be integrated	[1-4,7-11,16,18,21,23,25-29,32,34,36,42,45,47,53,58,60,62,66-70]
**Finding 13**	Primary health care level can identify some signs and symptoms before any health care level, favouring an early diagnosis	[1,2,4,8,21,23,27,32,35,38-40,42,44,45,47,52,58,61,69-71,80]
**Finding 29**	There is a need to establish and create specific-disease emergency protocols	[7,16,22,23,34-36,38,46,59,60,62,63,66,69,71,74-76]
**Technology**	Technology – the use of digital health solutions has been significantly impacting the diagnosis, treatment, and long-term follow-up of patients with rare diseases	[12,14,16,19,20,24,29,36,39,46,48,50,51,58,64,65,71,73,74]
**Finding 1**	Diagnoses of rare diseases is difficult	[18,21,25,35-38,48,52,57,58,72-74]
**Finding 32**	Patients and families experience an extensive number of consultations until a definitive diagnosis if a misdiagnose is not established priorly	[8,9,18,25,43,45,50,58,63,68]
**Finding 17**	The feeling of general practitioners to prioritize access to secondary and tertiary levels of care is relevant	[4,7,11,17,23,25,34,42,43,45,53,57]
**Finding 15**	There is a notable low interest in pharmaceutical industries in identifying new drugs for rare diseases because of their low prevalence	[4,14,15,25,33,35,45,49,58,61,77]
**Finding 31**	Primary health care system is the gateway for approaching and following up with patients with rare diseases	[8,11,15,26,30,34,39,47,59,69,71]
**Finding 23**	Healthcare providers usually seek information about rare diseases on websites and in peer-to-peer discussions	[4-6,8,15,22,23,29,53,61]
**Finding 27**	Continuous educational and training programs are primarily unknown by health care providers	[6,11,40,41,56,61,62,73,75,78]
**Finding 30**	Translating results from genetic counselling to “patients and parent’s language” should be endorsed and performed	[7,14,26,34,41,52,56,57,74,78]
**Finding 36**	The care for patients with rare diseases must be multidisciplinary	[11,14,21,30,32,58,60,67,73,75]
**Finding 35**	The prevalence of rare diseases is globally unknown and controversial	[10,18,25,43,45,50,58,63,68]
**Finding 16**	Lawmakers and multiple stakeholders are alert and engaged in improving the quality of life of patients with rare diseases	[4,14,19,35,42,46]
**Finding 21**	Family and advocacy agencies commonly request actions to offer an integrated and person-centred delivery of care	[4,23,32,34,35,42,67,72]
**Finding 3**	There is a remarkable difference in-between medical specialty	[25,40,50,53,54,58,76,78]
**Finding 18**	The global impact of rare diseases on patient’s lives is still unknown by health care providers	[4,-6,8,14,37,39,81]
**Finding 9**	There is a substantial economic impact caused by rare diseases in households, mostly related to expensive medications and diagnosis tests	[23,37,45,49,52,58,72]
**Finding 10**	Pre-natal and post-natal screening is essential to identify some rare diseases	[16,19,41,49,67,78]
**Finding 11**	Current health care systems settings do not allow a good experience for patients with rare diseases	[22,30,42,48,72,77]
**Finding 33**	Patients’ and family’s needs and concerns are not addressed by most health care professionals	[8,25,26,36,45,79]
**Finding 25**	Physicians fear delaying diagnosis because of lack of experience and believe that they have an active role in managing patients with rare diseases	[5,25,47,53,78]
**Finding 7**	Knowledge diffusion might help with diseases misunderstanding and lack of knowledge	[16,22,27,34,58]
**Finding 19**	Current state of medical education for approaching rare diseases is adequate	[4,10,22]
**Finding 37**	Judicialization is beneficial for patients with rare diseases	[49,55,77]
**Finding 14**	The complex knowledge needed for the management of rare diseases is incompatible with the primary health care	[2,81]
**Finding 22**	A critical complaint reported by health care providers is associated with the difficulty to information access about rare diseases	[54,81]
**Finding 26**	The impact of rare diseases on families stimulates health care providers to improve their knowledge	[5,23]
**Finding 28**	By anticipating and recognizing available regional interventions, health care providers can increase the delivery of care to patients with rare diseases	[7,30]
**Finding 34**	Strong medications are commonly prescribed for patients with rare diseases until a definite diagnose	[8,61]
**Finding 38**	Patients with rare diseases have significantly lower social interaction than patients without rare diseases	[48,79]
**Finding 39**	“Expert patients” might have a conflicting relationship with physicians during the management of their disorders	[23,29]
**Finding 5**	Fast identification of a rare disease results in the decrease of impact provoked by the disease and allow fast delivery of medical interventions	[47,19]
**Finding 2**	Nutritional follow-up of patients with rare diseases is crucial, particularly among patients with inborn errors of metabolism	[38]
**Finding 4**	The shortage of patients for research, including rare diseases patients, can be overcome with online recruiting platforms	[39]
**Finding 6**	Domestic (in-house) delivery of medical treatment should be considered in some rare diseases	[11]
**Finding 8**	Non-pharmacological interventions are effective and should be investigated	[30]
**Finding 24**	Social health care workers have a slightly increased experience than other health care providers in assessing and managing rare diseases	[5]
**Finding 40**	The roles of the primary health care level are numerous and should be strengthened	[60]

**Figure 2:**
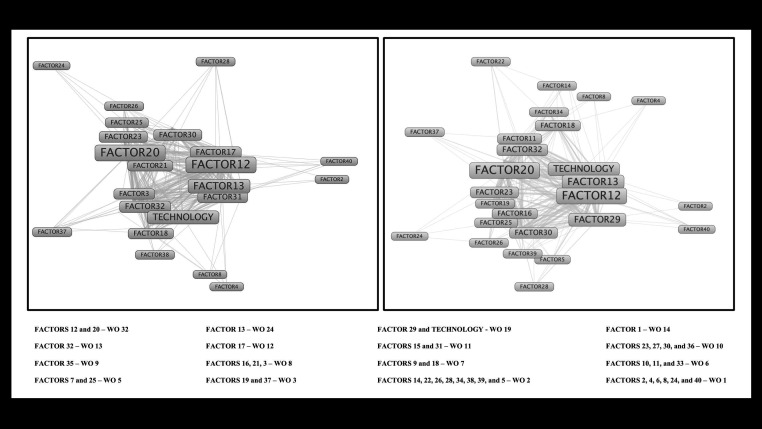
Relative frequency of occurrence of all clustered findings from included publication. WO – weight of occurence

Our thematic-based analysis yielded 41 principal codes and scope-related findings, as shown in **Table 3** and **Figure 2**. The four top-ranked results among our included emphasized the following: 1. the elevated health care professional-reported perception of insufficient or inadequate knowledge about rare diseases diagnosis and their management in primary care (weight of occurrence (WO) = 32, 34 conceptual links); 2. highlighted and advocated for the essential characteristics of the primary care system, including the promotion of collaborative care, delivery of patient- and family-centeredness care, integration and coordination of care, sustainability, better management of chronic diseases, and the longitudinal continuity of care and patient-family-professional relationship (WO = 30, 33 conceptual links); 3. primary care and its health care professionals' capacity to identify early signs and symptoms commonly reported and identified in patients with rare diseases (WO = 23, 31 conceptual links); and 4. the need to collaboratively establish emergency protocols for each patient based on their singularity and rare underlying disease, and the need to standardize medical procedures and treatments for these patients (WO = 18, 27 conceptual links). The vast number of consultations until the final diagnosis (usually expensive; WO = 13, 24 conceptual links), the importance of prioritization access to specialists and philanthropic / supporting organizations (WO = 12, 27 conceptual links), and the low interest of pharmaceutical industries in identifying new pharmacological interventions (WO = 10, 27 conceptual links) were also significant findings observed among included studies. The list of all categorical results coded and clustered in our analysis can be accessed in **Figure 2**.

**Table 3:** Descriptive mathematical evaluation of identified factors and their in-map representation

Finding	X	Y	Cluster	Links	Total link strength	Weight of occurrences (WO)
**Factor 12**	-0.0709	0.0246	5	33	138	32
**Factor 20**	0.2859	-0.0323	2	34	130	32
**Factor 13**	0.0826	0.1767	6	31	99	24
**Factor 29**	-0.7848	-0.1992	6	28	74	19
**Technology**	-0.0346	0.218	2	25	55	19
**Factor 1**	-0.6885	-0.0189	6	23	60	14
**Factor 32**	0.2806	0.246	1	24	64	13
**Factor 17**	-0.1431	-0.1785	7	27	68	12
**Factor 15**	0.0089	-0.2594	4	27	61	11
**Factor 31**	-0.097	0.5413	1	25	52	11
**Factor 23**	0.7086	-0.3081	3	24	53	10
**Factor 27**	-0.2173	0.4197	5	18	32	10
**Factor 30**	-0.4871	-0.2475	7	21	39	10
**Factor 36**	-0.7549	0.367	5	24	43	10
**Factor 35**	-0.3745	-0.3559	7	18	43	9
**Factor 16**	0.2098	-0.249	4	17	35	8
**Factor 21**	0.1515	-0.5726	3	21	45	8
**Factor 3**	0.4792	0.1978	2	20	39	8
**Factor 18**	0.6282	0.6517	2	22	34	7
**Factor 9**	0.039	-0.5233	4	24	42	7
**Factor 10**	-0.0175	-0.39	4	16	22	6
**Factor 11**	-0.4075	0.0274	1	20	23	6
**Factor 33**	-0.0759	0.3735	1	19	36	6
**Factor 25**	0.7296	-0.4361	3	18	27	5
**Factor 7**	-0.3502	-0.494	8	19	29	5
**Factor 19**	0.1806	-0.4801	8	13	17	3
**Factor 37**	-0.1992	-0.5305	4	5	6	3
**Factor 14**	11.184	0.9388	2	5	5	2
**Factor 22**	10.979	0.8953	2	5	6	2
**Factor 26**	0.9688	-0.7318	3	12	14	2
**Factor 28**	-0.9952	-0.622	7	6	7	2
**Factor 34**	0.5016	0.5847	1	10	13	2
**Factor 38**	-0.5205	0.3038	1	5	5	2
**Factor 39**	0.7243	-0.7233	3	11	13	2
**Factor 5**	0.434	-0.2874	4	7	8	2
**Factor 2**	-13.565	-0.1049	6	3	3	1
**Factor 24**	1.113	-0.732	3	4	4	1
**Factor 4**	0.3963	0.9528	2	4	4	1
**Factor 40**	-1.399	0.3423	5	3	3	1
**Factor 6**	-0.6057	0.7424	5	5	5	1
**Factor 8**	-0.5586	0.4732	1	4	4	1

The development and incorporation of digital health technologies (mainly computerized clinical decision support systems) were endorsed and suggested in several publications [20,22,24,30,34,43,45,52,54,56,57,65,71,72,78,80,81,90]. The use of newcomer technologies for early diagnosis, delivery of care, and long-lasting follow-up of patients with rare diseases was indicated in 16 publications. Mainous and colleagues suggested a significant improvement in managing sick cell disease patients in primary care following the implementation of clinical decision support appliances [57]. Additionally, Ehsani-Moghaddam et al. launched a Näive Bayes classification algorithm using symptoms and clinical data of patients with mucopolysaccharidosis type II registered in the Canadian Primary Sentinel Surveillance Network [30]. The classifier has effectively assisted physicians at the primary care level in diagnosing the syndrome and allowing optimal patient long-term management. Likewise, Buendia et al. developed and implemented a primary care rare disease case-finding tool based on data from pseudo-anonymized electronic medical records data [20]. This study validated that a rare disease case-finding software could be appropriately designed and implemented following patients' data security recommendations. Moreover, it demonstrated the feasibility of using patients' phenotypes documented within primary care electronic records as the basis for case algorithms. In addition, some records superficially pinpointed the benefits of such digital health technologies, improving interprofessional communication (in particular between the specialist and the primary care physician), democratizing access to health-related information, and facilitating the obtention of technical information for health care professionals. However, as stressed by Terry and Krokosky, there is an ongoing concern about these digital solutions as the media tends to show digital solutions with commercial denotations [81]. Interestingly, one study evidenced the inefficiency of computer-based predictive model alerts to physicians in diagnosing rare diseases [71].

Government agencies have also suggested the creation of tools and regulations and the use of digital health technologies for addressing cases of rare diseases assisted in the primary health care sector [19,22,39,52,65,68,72]. In 2013, the Department of Healthcare of Buenos Aires (Argentina) endorsed a group of normative guidelines that focused on developing health policies and tools digitally structured for accurate diagnosis and local investigation of rare diseases [22]. Similarly, as stated by García-Ribes et al. [40] and Morales-Piga et al. [65], the use of online systems and assistance protocols in Spain has been well-established for diagnosis support, information provision, coordination of care, and delivery of epidemiological data to register centres. In the Brazilian scenario, specialized care coordination is provided by laws that focus on health promotion, early diagnosis, appropriate and timely secondary and tertiary care referral, and humanized and patient-centred care [68]. One study reported the improvement and creation of an online register connecting the primary and secondary care levels, which might provide better patient data due to the completeness of data [72].

## DISCUSSION

This qualitative synopsis and thematic-based assessment highlighted several connected components between primary care and rare diseases. Eighty publications were published within the past 18 years, indicating an increasing interest of researchers and other stakeholders (eg, government and patient support organizations) in understanding the importance of primary care as a system that delivers holistic, coordinated, and long-lasting care. However, a focused and in-depth evaluation of a determined disease could not be carried out as most publications regarded “rare diseases in general” or several other targeted diseases. Eighteen publications suggested the importance of digital health technologies in diagnosing rare diseases promptly to enhance patients' and caregivers' experiences throughout the natural course of the disease. Most importantly, health care professionals stressed the insufficient knowledge of managing patients with rare diseases (stating that the topic was rarely approached in universities). Nevertheless, despite limited training and knowledge regarding these patients' diagnosis, management, and long-term follow-up, these professionals ensured the power and high effectiveness attributed to the primary health care system to guarantee the delivery of essential health elements for individuals living with rare diseases. Furthermore, the primary health care sector's ability and responsibility to identify signs and symptoms presented in daily medical consultations were notably observed among included publications. The least frequently generated codes related to the existence and effectiveness of non-pharmacological interventions (integrative health practices) in treating patients with rare diseases, the importance of strengthening the primary health care system, and the potential advantages social health care workers have over other professionals in dealing with individuals living with rare diseases.

As suggested in our findings, empowering the primary health care system positively affects patients with rare diseases. However, it might also improve the application intelligibility of this system in more complex medical conditions and emergencies, such as pandemics, health emergencies, and natural catastrophes [35,36]. The primary health care system can coordinate local and regional networks, address the population's needs, and integrate care, prevention, promotion, and education. In addition, the system significantly improves overall health care performance by decreasing governmental expenditure and offering a holistic delivery of care [40]. However, considering the global and growing rhetoric focusing on dismantling the primary health system structure, it is essential to assess the risks of these attempts to organize counteracting plans to strengthen the system. Likewise, educational programs (such as television or radio communications as well as social media publishing) focusing on the beneficial relationship between the primary health care system, rare diseases, and other conditions should also be part of core actions to enhance the system’s popularity, acceptability, and awareness of efficacy, always considering each population's cultural, social, and financial singularities. Moreover, courses and update workshops for health care professionals, discussing features such as the management, diagnosis, treatment, and long-term follow-up in the primary health care system, should also be encouraged by municipalities and governments elsewhere. Notably, working with multiple health-related specialties and teams (emphasizing the importance of multidisciplinary teams) could effectively improve the client and professional experience with the system.

The singularities of each health care system (considering social, political, and economic variables) and the particularities of different diseases directly affect rare disease management and how patients experience care delivery within the health care system. Although health care systems in several places have increased their focus on a better understanding of the processes in that patients with rare diseases are involved, some features and concepts are still misunderstood, outdated, or disregarded by health providers and local administrative agencies, resulting in totally different perception of an identical condition [41,55,60]. Notably, worldwide, health care systems still struggle to develop a valuable and comparable notification system for registering patients with rare diseases diagnosed. This issue creates a profound gap in the understandability of the same condition identified in a different location as the comparison of data are hindered by the contrasting information about patients and confounders inherent in the data collection period [41,62,64,70]. This problem becomes even worse when health care professionals do not see the importance of establishing and assessing regional or national indicators associated with a rare disease, as data collection is usually simply related to “random and unnecessary” obtention of epidemiological indexes for the creation of statistical analyses. However, it is worthwhile mentioning that understanding the prevalence and incidence of a particular rare disease within a community not only guarantees cost-efficient resource use but also allows the foundation of a better framework of what needs this specific population will need in the long run. Therefore, to tackle the differences between health care systems worldwide, a structural change in the health care system’s format to deal with rare diseases is needed to create equivalent and comparable data and overcome interregional disparities.

From a theoretical perspective, the achievement of the United Nations’ Sustainable Development Goals (SDGs) is directly affected by countries’ health care systems and institutionalized strategies for delivering care. Although a remarkable global improvement in population health outcomes has been observed over the last decades, the divisive private-based health care system will continue to set millions of people apart from a less unequal and unhealthy life. To note, it is inevitable not to assimilate the primary health care approach and public health care-based systems, as both processes are rooted in the capacity to respond equitably, inclusively, and cost-effectively to the health need of citizens. As our findings show, the primary health care approach of care positively affects health outcomes for the rarest diseases, from diagnosis to palliative care, making the creation of initiatives against the public health care system dismantling rhetoric critical. As advocated by the American Academy of Family Physicians and National Health System England, the call for action poses a significant starting point to counteract the long-dated initiatives to set primary and public care apart [99,100]. However, more is needed from a provider, institution, community, region, and national perspective.

Enhancing the number of high-quality genetics, experimental, and clinical research devoted to rare diseases, or improving the knowledge of the impact of primary care services for rare diseases are cornerstones of scientific improvement toward achieving Universal Health Coverage (UHC). Our data clearly suggested that several publications evidenced the minimum interest of pharmaceutical industries to identify and formulate new pharmacological therapies because of the low profitability and limited growth capacity attributed to rare diseases. Ultimately, the little research and production of new drugs directly hinder the achievement of SDG Goal 3 as highly advanced technologies and qualified professionals are not involved in improving health quality. Indeed, no substantial evidence has shown that pharmaceutical companies have been actively developing new drugs in a broad and non-selective way. For instance, although Pzifer has taken measures to create pharmacological alternatives for various diseases, 104 discovery projects have been reported as of July 28, 2022 [101]. Of those, only 11 compounds cover patients with rare diseases. Nevertheless, these “research discoveries and development” might be inflated as in-house development has been recorded to be relatively low [102]. Therefore, third parties as universities and health educational institutions play an essential role in fulfilling the current comprehension gaps, as pharmaceutical industries do not offer outstanding improvements for patients at the pace needed, specifically for rare diseases. Likewise, as far as recent advances in mRNA and DNA technology are concerned (making the new era of genetics therapy a reality), exploring this new (and potentially unknown) area of expertise is of utmost importance to improve the general delivery of care to patients with rare disorders, regardless the level of care.

Reducing social and economic disparities (SDG Goal 10) among patients (and their families) diagnosed with rare diseases also has particular importance in the 2030 Agenda for Sustainable Development. Rare disease patients regularly face a shortage of health resources, precarious education, and a structured family core, which combined marginalize these patients over the years. Our data showed that the financial impact of rare diseases on the family budget is significant, driven mainly by expensive medications, especially biologics. Based on data reported by Gimenez-Lozano et al., approximately 58% of families with a rare disease-confirmed case reported not being able to afford adjuvant therapies [42]. Moreover, the disease brings not only a financial impact but also a psychological and emotional burden [42]. Thus, counteracting actions to reduce inequality should focus on active advocacy for the inclusion of rare disease patients in all social spheres, either by using legislative changes or through supportive public programs, as well as strengthening the primary health care system and its core principles.

Various modalities of digital health solutions were reported in included studies. Despite these interventions' high efficiency, accuracy, scalability, and safety, several components should be considered before creating and implementing these systems. First, the design of digital health technologies should identify end-users' needs, perceived utility, and ability to use such technologies, particularly for physicians who frequently do not have any prior preparation for utilizing health technologies in most medical universities. Additionally, understanding barriers to the success of the intervention must analyse inherent infrastructure and technical difficulties experienced by users, health care providers' personal and psychological issues, legal and ethical variables, and cultural, social, and geopolitical factors. Therefore, the involvement of providers and other social actors in creating and employing digital health technologies, the evaluation of providers' willingness to use the technologies, and the existence of institutional and governmental incentives are of utmost importance.

The creation of standardized and instructive emergency protocols in the event of sudden decompensation or complication was interestingly ranked as the fourth most frequent concept among included publications. Due to inherent features of the primary care consultation approach (eg, longer consultation times and stronger provider-patient relationship), preparing these simple materials at the primary care level is timely and efficient. Not only does preparing these materials seem critical, but regular health assessments and preventive clinical actions are fundamental. For instance, based on regular lower respiratory tract cultures check-ups performed at the primary care level, previously generated data can guide physicians in managing acute pulmonary exacerbations in patients with cystic fibrosis, essentially due to chronic colonization of some bacteria (eg, *Pseudomonas aeruginosa* or *Staphylococcus aureus*). We strongly recommend preparing such medical instruments early in disease recognition as information obtention in emergency conditions is usually chaotic and challenging. Therefore, there is no better and more controlled place than the primary health care offices to leverage guidelines about rare disease complications and unexpected clinical presentations. To the best of our knowledge, no previous template of an “Emergency Protocol for Patients Living with Rare Diseases” has been published elsewhere. However, we endorse that basic information containing the patient’s medical history and background, diagnosis, medications on use, allergies, and description of the acute complication must be written in these protocols. Moreover, intervention contra-indication and dangerous drug-to-drug interactions should also be inserted in these instructive and informative materials. In Section 3 in the **Online Supplementary Document**, standardized, editable, and adaptable material is shared for health care providers working in primary health care facilities.

Relying on information about the diagnosis, management, and treatment in general websites and by peer-to-peer consultation was frequently identified as the leading knowledge resource by health care professionals. Nevertheless, particular attention should be given to rare disease-related information resources as multiple platforms are not official, based on high-quality evidence, and are subjective to clinicians’ opinions and expertise. Recently, evidence-based reports have suggested the high prevalence of misinformation and disinformation on online pages with health content and digital devices, which significantly reduces the trustworthiness of unofficial health resources [103]. As emphasized in some included publications, accessing information on rare diseases is complex, and consulting the first Google search page might be a reality. However, based on the data retrieved, we reiterate the existence of several credited and expert-authored online platforms available for health care professional consultation, which properly guides professionals during any delivery of assistance for patients with rare diseases.

Our study has several strengths. First, we strictly followed international and standard methodological recommendations for evidence-based studies. Additionally, we included a relevant number of patients and health care professionals from the primary health care sector, which allows us to upgrade the general certainty of the evidence herein provided. To note, although the creation of a meta-analytic assessment was not feasible, we created a thematic analysis based on the occurrence of similar semantic terms or sentences verified among included publications. This evaluation process is highly subjective and the displayed mathematical results of the weight of occurrences might be affected by this subjectivity. However, we strictly attained critical methodological concepts, including criteria of credibility, transferability, dependability, and confirmability throughout the project execution. Significant limitations might be associated with patients' and health care professionals' selection bias in included studies, the lack of comprehensive description of demographic and epidemiological data from individuals being addressed among included studies, and the limited number of studies outside the Americas and European Regions. This could directly hinder the generalizability of our findings to other scenarios.

## CONCLUSION

Our study highlights the commonly reported concepts when merging “primary healthcare” and “rare diseases”. We observed relevant evidence suggesting the importance of primary health care level of care in managing and supporting individuals living with rare diseases. Our study suggests the strengths and weaknesses within this relationship and shows future perspectives which decision-makers, technology developers, and health care providers must follow to improve the quality of care delivered to patients with rare diseases.

## Additional material


Online Supplementary Document

## References

[1] Huertas-Quintero JA Losada-Trujillo N Cuellar-Ortiz DA Velasco-Parra HM Hypophosphatemic Rickets in Colombia: A Prevalence-Estimation Model in Rare Diseases. 2018. Lancet Reg Health Am. 2021;7:100131. 10.2139/ssrn.3854741 36777652 PMC9904046

[2] Nguengang Wakap S Lambert DM Olry A Rodwell C Gueydan C Lanneau V Estimating cumulative point prevalence of rare diseases: analysis of the Orphanet database. Eur J Hum Genet. 2020;28:165-73. 10.1038/s41431-019-0508-0 31527858 PMC6974615

[3] Black MM Walker SP Fernald LCH Andersen CT DiGirolamo AM Lu C Early childhood development coming of age: science through the life course. Lancet. 2017;389:77-90. 10.1016/S0140-6736(16)31389-7 27717614 PMC5884058

[4] United Nations. THE 17 GOALS | Sustainable Development. Available: https://sdgs.un.org/goals. Accessed: 18 October 2022.

[5] World Health Organization. Primary health care. Available: https://www.who.int/health-topics/primary-health-care. Accessed: 18 October 2022.

[6] Kruk ME Gage AD Arsenault C Jordan K Leslie HH Roder-DeWan S High-quality health systems in the Sustainable Development Goals era: time for a revolution. Lancet Glob Health. 2018;6:e1196-252. 10.1016/S2214-109X(18)30386-3 30196093 PMC7734391

[7] Arksey H O’Malley L Scoping studies: towards a methodological framework. Int J Soc Res Methodol. 2005;8:19-32. 10.1080/1364557032000119616

[8] Tricco AC Lillie E Zarin W O’Brien KK Colquhoun H Levac D PRISMA Extension for Scoping Reviews (PRISMA-ScR): Checklist and Explanation. Ann Intern Med. 2018;169:467-73. 10.7326/M18-0850 30178033

[9] Orphanet. ORPHANET - Rare diseases. Available: https://www.orpha.net/consor/cgi-bin/index.php. Accessed: 18 October 2022.

[10] Acevedo Gragera A Fernández Rojas J Salas Campo E Progeria del adulto (síndrome de Werner). Seguimiento de 2 casos desde Atención Primaria. SEMERGEN. Soc Esp Med Rural Gen. 2006;32:410-4.

[11] Amaral VFM Martins AAS Quando a preguiça é sinônimo de doença - um caso de doença de McArdle. Rev Bras Med Fam Comunidade. 2016;11:1-6. 10.5712/rbmfc11(38)1277

[12] Avellaneda A Izquierdo M Torrent-Farnell J Ramón JR Enfermedades raras: enfermedades crónicas que requieren un nuevo enfoque sociosanitario. An sist sanit Navar. 2007;30:177–90. 10.4321/S1137-66272007000300002 17898813

[13] Avellaneda Fernández A Izquierdo Martínez M Luengo Gómez S Arenas Martín J Ramón JR Need for primary care training in rare diseases. Aten Primaria. 2006;38:345-8. 17173799 10.1157/13093372 PMC7669153

[14] Avellaneda Fernández A Pérez Martín A Pombo Allés G Gutiérrez Delgado E Izquierdo Martínez M Perception of rare diseases by the primary care physicians. Semergen. 2012;38:421-31. 23021574 10.1016/j.semerg.2012.02.011

[15] Beck N Applegate C Elements of genetic counseling for inborn errors of metabolism. Transl Sci Rare Dis. 2020;4:197-208. 10.3233/TRD-190044

[16] Bedin K Silva MJC Guerra PH Friestino JKO Doenças raras e práticas de saúde coletiva: relato de experiência na formação médica. Rev APS. 2021;24:780-7.

[17] Benson M Albanese A Bhatia K Cavillon P Cuffe L Konig K Development of a patient journey map for people living with cervical dystoni. Eur J Neurol. 2022;29:683. 10.1186/s13023-022-02270-4 PMC8935780 35313909

[18] Boffin N Swinnen E Wens J Urbina M Heyden JVD Casteren VV General practice care for patients with rare diseases in belgium. A cross-sectional survey. Int J Environ Res Public Health. 2018;15:1180. 10.3390/ijerph15061180 29874870 PMC6025074

[19] Brasil. Ministério da Saúde. Coordenação Geral de Média e Alta Complexidade. Diretrizes para atenção integral às pessoas com doenças raras no Sistema Único de Saúde SUS. 2014. Available: http://conitec.gov.br/images/Protocolos/Diretrizes_Atencao-DoencasRaras.pdf. Accessed: 18 October 2022.

[20] Buendia O Shankar S Mahon H Toal C Menzies L Ravichandran P Is it possible to implement a rare disease case-finding tool in primary care? A UK-based pilot study. Orphanet J Rare Dis. 2022;17:54. 10.1186/s13023-022-02216-w 35172857 PMC8848904

[21] Bueno EG Ruano García M Guerra de los Santos JM Montero Vásquez I Conocimientos médicos sobre enfermedades raras por parte de los profesionales de la salud. Salud(i)ciencia (Impresa). 2015;21:604–9.

[22] Buenos Aires. Ministerio de Salud. Subsecretaría de Coordinación y Atención de la Salud. Dirección Provincial de Atención Primaria de la Salud. Dirección de Patologías Prevalentes. Cuidado integral de personas con enfermedades raras. enfermedades raras. La APS renovada en la Provincia de Buenos Aires. 2013;v.4

[23] Byrne N Turner J Marron R Lambert DM Murphy DN O’Sullivan G The role of primary care in management of rare diseases in Ireland. Ir J Med Sci. 2020;189:771-6. 10.1007/s11845-019-02168-4 31933130 PMC7363724

[24] Canadian Organization for Rare Disorders. Now is the Time: A Strategy for Rare Diseases is a Strategy for all Canadians. Available: https://www.raredisorders.ca/content/uploads/CORD_Canada_RD_Strategy_22May15.pdf. Accessed: 18 October 2022.

[25] Castro-Rodríguez E Azagra R Gómez-Batiste X Povedano M [Amyotrophic lateral sclerosis (ALS) from the perspective of Primary Care. Epidemiology and clinical-care characteristics]. Aten Primaria. 2021;53:102158. 34509895 10.1016/j.aprim.2021.102158 PMC8435918

[26] Derayeh S Kazemi A Rabiei R Hosseini A Moghaddasi H National information system for rare diseases with an approach to data architecture: A systematic review. Intractable Rare Dis Res. 2018;7:156-63. 10.5582/irdr.2018.01065 30181934 PMC6119672

[27] Domínguez Enríquez JP Navarrete Socasi D Auquillas Cajamarca B Cusco Cuzco C Neurofbromatosis: reporte de un caso diagnosticado en atención primaria. VozAndes. 2013;24:75-8.

[28] Druschke D Krause F Müller G Scharfe J Hoffmann GF Schmitt J Potentials and current shortcomings in the cooperation between German centers for rare diseases and primary care physicians: results from the project TRANSLATE-NAMSE. Orphanet J Rare Dis. 2021;16:494. 10.1186/s13023-021-02106-7 34819135 PMC8611963

[29] Dudding-Byth T A powerful team: the family physician advocating for patients with a rare disease. Aust Fam Physician. 2015;44:634-8. 26488040

[30] Ehsani-Moghaddam B Queenan JA MacKenzie J Birtwhistle RV Mucopolysaccharidosis type II detection by Naïve Bayes Classifier: An example of patient classification for a rare disease using electronic medical records from the Canadian Primary Care Sentinel Surveillance Network. PLoS One. 2018;13:e0209018. 10.1371/journal.pone.0209018 30566525 PMC6300265

[31] Elliott E Zurynski Y Rare diseases are a “common” problem for clinicians. Aust Fam Physician. 2015;44:630-3. 26488039

[32] Esteban Bueno G Ruano García M García Luna P Motero Vázquez I. El médico de familia ante la esclerosis lateral Amiotrófica. Rev clín med fam. 2016;9:46–9.

[33] Esteban-Bueno G Díaz-Anadón LR Rodríguez González A Navarro Cabrero M Berenguel Hernández AM [Genetic protocol in primary care for rare diseases: Wolfram syndrome as a prototype]. Aten Primaria. 2022;54:102285. 10.1016/j.aprim.2022.102285 35307613 PMC8931343

[34] Evans W Buendia O Toal C Ravichandran P Menzies L Rare disease case-finding using a digital tool in UK primary care -a pilot study. Eur J Hum Genet. 2022;30:464-5. 10.1186/s13023-022-02216-w PMC8848904 35172857

[35] Evans WRH Rafi I Rare diseases in general practice: Recognising the zebras among the horses. Br J Gen Pract. 2016;66:550-1. 10.3399/bjgp16X687625 27789486 PMC5072891

[36] Fabrizzio GC Gonçalves Júnior E da Cunha KS Kahl C dos Santos JLG Erdmann AL Gestão do cuidado de um paciente com Doença de Devic na Atenção Primária à Saúde. Rev Esc Enferm USP. 2018;52:e03345. 10.1590/s1980-220x2017024603345 30088542

[37] Falah N Umer A Warnick E Vallejo M Lefeber T Genetics education in primary care residency training: satisfaction and current barriers. BMC Prim Care. 2022;23:156. 10.1186/s12875-022-01765-0 35718772 PMC9208192

[38] Francisco R Magrinyà P Torrent J Jiménez J Franch L Guarga À Catalonia’s care model for rare diseases (Spain). Orphanet J Rare Dis. 2018;13:167.

[39] Gammie T Lu CY Babar ZU-D Access to Orphan Drugs: A Comprehensive Review of Legislations, Regulations and Policies in 35 Countries. PLoS One. 2015;10:e0140002. 10.1371/journal.pone.0140002 26451948 PMC4599885

[40] Garcia Ribes M The diagnosis of rare diseases in the primary care clinic: Dismantling the myth. Aten Primaria. 2013;45:338-40. 10.1016/j.aprim.2013.01.010 23541112 PMC6985481

[41] García-Ribes M Ejarque I Arenas E Martín V New challenges: General practitioners faced with “rare diseases.”. Aten Primaria. 2006;37:369-70. 16789339 10.1157/13087370 PMC7679924

[42] Gimenez-Lozano C Páramo-Rodríguez L Cavero-Carbonell C Corpas-Burgos F López-Maside A Guardiola-Vilarroig S Rare Diseases: Needs and Impact for Patients and Families: A Cross-Sectional Study in the Valencian Region, Spain. Int J Environ Res Public Health. 2022;19:10366. 10.3390/ijerph191610366 36012000 PMC9408677

[43] González-Lamuño D Couce ML Amor Bueno M Aldámiz-Echevarría L When rare diseases become urgent: inborn errors of metabolism in primary care. Aten Primaria. 2009;41:221–6. 19328600 10.1016/j.aprim.2008.07.013 PMC7021945

[44] Grupo de Trabajo SEMFyC Genética Clínica y Enfermedades Raras “Abordando las Enfermedades Raras desde la consulta de Atención Primaria: si se quiere, se puede”. Rev clín med fam. 2013;6:32–6. 10.4321/S1699-695X2013000100006

[45] Gupta S Bayoumi AM Faughnan ME Rare lung disease research: Strategies for improving identification and recruitment of research participants. Chest. 2011;140:1123-9. 10.1378/chest.11-1094 22045877

[46] Hariyan T Kinash M Kovalenko R Boyarchuk O Evaluation of awareness about primary immunodeficiencies among physicians before and after implementation of the educational program: A longitudinal study. PLoS One. 2020;15:e0233342. 10.1371/journal.pone.0233342 32470021 PMC7259605

[47] Hayward J Bishop M Rafi I Davison V Genomics in routine clinical care: what does this mean for primary care? Br J Gen Pract. 2017;67:58-9. 10.3399/bjgp17X688945 28126856 PMC5308090

[48] Iskrov G Stefanov R Ferrelli RM Health systems for rare diseases: financial sustainability. Ann Ist Super Sanita. 2019;55:270-5. 31553322 10.4415/ANN_19_03_13

[49] Jo A Larson S Carek P Peabody MR Peterson LE Mainous AG Prevalence and practice for rare diseases in primary care: A national cross-sectional study in the USA. BMJ Open. 2019;9:e027248. 10.1136/bmjopen-2018-027248 30940763 PMC6500220

[50] Kim D Eshtiaghpour D Alpern J Datta A Eysselein VE Yee HF Access to primary care is associated with better autoimmune hepatitis outcomes in an urban county hospital. BMC Gastroenterol. 2015;15:91. 10.1186/s12876-015-0318-y 26215250 PMC4517362

[51] Knight AW Senior TP The common problem of rare disease in general practice. Med J Aust. 2006;185:82-3. 10.5694/j.1326-5377.2006.tb00477.x 16842062

[52] Koch V Grindler C Hira A Carneiro-Sampaio M Dora (doencas raras) project: A proposal for an integrated management of rare diseases in the state of Sao Paulo, Brazil. Mol Syndromol. 2012;2:269-70.

[53] Loio M Salgueiro A Cruz H Dermatomiosite juvenil papel do médico de família na abordagem de uma doença rara. Rev Bras Med Fam Comunidade. 2017;12:1-8. 10.5712/rbmfc12(39)1418

[54] Lopes MT Koch VH Sarrubbi-Junior V Gallo PR Carneiro-Sampaio M Difficulties in the diagnosis and treatment of rare diseases according to the perceptions of patients, relatives and health care professionals. Clinics (São Paulo). 2018;73:e68. 10.6061/clinics/2018/e68 29641803 PMC5866403

[55] Luz G dos S Silva MRS da, DeMontigny F. Doenças raras: itinerário diagnóstico e terapêutico das famílias de pessoas afetadas. Acta Paul Enferm. 2015;28:395-400. 10.1590/1982-0194201500067

[56] Maggi L Vita G Marconi E Taddeo D Davì M Lovato V Opportunities for an early recognition of spinal muscular atrophy in primary care: a nationwide, population-based, study in Italy. Fam Pract. 2023;40:308-313. 10.1093/fampra/cmac091 35950319

[57] Mainous AG Carek PJ Lynch K Tanner RJ Hulihan MM Baskin J Effectiveness of clinical decision support based intervention in the improvement of care for adult sickle cell disease patients in primary care. J Am Board Fam Med. 2018;31:812-6. 10.3122/jabfm.2018.05.180106 30201679 PMC6153439

[58] Martínez-Sabater A Sancho-Cantus D Arnold-Chiari malformation and syringomyelia in primary care. A case report. Semergen. 2012;38:331-4. 10.1016/j.semerg.2011.09.007 23544780

[59] McClain MR Cooley WC Keirns T Smith A A survey of the preferences of primary care physicians regarding the comanagement with specialists of children with rare or complex conditions. Clin Pediatr (Phila). 2014;53:566-70. 10.1177/0009922814528035 24671871

[60] Mehta A Belmatoug N Bembi B Deegan P Elstein D Göker-Alpan Ö Exploring the patient journey to diagnosis of Gaucher disease from the perspective of 212 patients with Gaucher disease and 16 Gaucher expert physicians. Mol Genet Metab. 2017;122:122-9. 10.1016/j.ymgme.2017.08.002 28847676

[61] Melo DG de Paula PK de Araujo Rodrigues S da Silva de Avó LR Germano CMR Demarzo MMP Genetics in primary health care and the National Policy on Comprehensive Care for People with Rare Diseases in Brazil: opportunities and challenges for professional education. J Community Genet. 2015;6:231-40. 10.1007/s12687-015-0224-6 25893505 PMC4524835

[62] Melo DG Germano CMR Porciúncula CGG Paiva IS de Neri JI da CF Avó LR da S de Qualificação e provimento de médicos no contexto da Política Nacional de Atenção Integral às Pessoas com Doenças Raras no Sistema Único de Saúde (SUS). Interface (Botucatu, Online). 2017;21.

[63] Menon D Clark D Stafinski T Reimbursement of Drugs for Rare Diseases through the Public Healthcare System in Canada: Where Are We Now? Healthc Policy. 2015;11:15-32. 10.12927/hcpol.2015.24360 26571466 PMC4748363

[64] Ministry of Health and Long-Term Care Government of Ontario. Old Foes and New Threats - Ontario’s Readiness for Infectious Diseases - 2012 Annual Report of the Chief Medical Officer of Health of Ontario to the Legislative Assembly of Ontario - Dr. Arlene King, Chief Medical Officer of Health - Ministry Reports - Publications - Public Information - MOHLTC. Government of Ontario, Ministry of Health and Long-Term Care. Available: https://www.health.gov.on.ca/en/common/ministry/publications/reports/rare_diseases_2017/default.aspx. Accessed: 18 October 2022.

[65] Morales-Piga A García Ribes M Arribas Álvaro P Casado Álvaro C Posada De La Paz M Bachiller-Corral J Is there a place in primary care for rare diseases? the case of fibrodysplasia ossificans progressiva. Aten Primaria. 2013;45:324-8. 10.1016/j.aprim.2012.11.014 23369643 PMC6985523

[66] Murphy PZ Thomas J McClelland TP Pain Management of Budd Chiari Syndrome in the Primary Care Setting: A Case Study. Innov Pharm. 2021;12:10.24926/iip.v12i2.3906. 10.24926/iip.v12i2.3906 34345504 PMC8326689

[67] Núcleo de Telessaúde Santa Catarina. Como manejar pessoas com doenças raras na APS? 2015. Available: https://aps-repo.bvs.br/aps/como-manejar-pessoas-com-doencas-raras-na-aps/?utm_source=rss&utm_medium=rss&utm_campaign=como-manejar-pessoas-com-doencas-raras-na-aps. Accessed: 18 October 2022.

[68] Núcleo Telessaúde Estadual do Rio Grande do Sul. Schuler-Faccini L, Sociedade Brasileira de Genética Médica, Serviço de Genética Médica do Hospital das Clínicas de Porto Alegre, Departamento de Genética da Universidade Federal do Rio Grande do Sul, Programa Nacional de Telessaúde Brasil Redes. Anomalias congênitas ou de manifestação tardia - Portaria GM/MS n^o^ 199/2014 Eixo I Grupo 1. 2015. Available: https://ares.unasus.gov.br/acervo/handle/ARES/2087. Accessed: 18 October 2022.

[69] Ortega Calvo M. Las enfermedades raras como paradigma científico en atención primaria. Cuad gest prof aten prim (Ed. Impr.). 2004;10:41–4.

[70] Ortega Calvo M García De La Corte F Iglesias Bonilla P Rare diseases at a primary care facility. An Med Interna. 2007;24:535-8. 18275262 10.4321/s0212-71992007001100005

[71] Pearce F Lanyon PC Watts RA Can prediction models in primary care enable earlier diagnosis of rare rheumatic diseases? Rheumatology (Oxford). 2018;57:2065-6. 10.1093/rheumatology/kex508 29462470

[72] Pericleous M Kelly C De Lusignan S Ala A A novel UK regional real-world registry for primary biliary cholangitis (PBC) allowing data linkage from primary and secondary care. United European Gastroenterol J. 2018;6:A151-2.

[73] Ramalle-Gómara E Domínguez-Garrido E Gómez-Eguílaz M Marzo-Sola ME Ramón-Trapero JL Gil-De-Gómez J Education and information needs for physicians about rare diseases in Spain. Orphanet J Rare Dis. 2020;15:18. 10.1186/s13023-019-1285-0 31952528 PMC6969468

[74] Reimann A Bend J Dembski B Patient-centred care in rare diseases. A patient organisations’ perspective. Bundesgesundheitsblatt Gesundheitsforschung Gesundheitsschutz. 2007;50:1484-93. 10.1007/s00103-007-0382-8 18026887

[75] Rodríguez de Mingo E Riofrío Cabeza S Villa Albuger T Velasco Blanco M Arteritis de Takayasu y trombosis venosa cerebral: a propósito de un caso. SEMERGEN. Soc Esp Med Rural Gen. 2014;40:e18-22. Ed impr. 10.1016/j.semerg.2012.10.003 24468302

[76] dos Santos CS Kishi RGB da Costa DLG da Silva DSD Narciso TRF da Silva de Avó LR Identificação de doenças genéticas na Atenção Primária à Saúde: experiência de um município de porte médio no Brasil. Rev Bras Med Fam Comunidade. 2020;15:2347. 10.5712/rbmfc15(42)2347

[77] Senior T Knight A Rare diseases: a role for primary care. Lancet. 2008;372:890. 10.1016/S0140-6736(08)61394-X 18790307

[78] Siderius L Beacher S Goossen W Care for the child with a rare disease: A joint venture. Arch Dis Child. 2012;97:A138. 10.1136/archdischild-2012-302724.0471

[79] de Silva KCS Fernandes LTB de Oliveira MVM Braga TC de Silva KL Desafios de cuidadores familiares de crianças e adolescentes com Epidermólise Bolhosa. Ciênc cuid saúde. 2020;19:e50427.

[80] Stroes E Moulin P Parhofer KG Rebours V Löhr J-M Averna M Diagnostic algorithm for familial chylomicronemia syndrome. Atheroscler Suppl. 2017;23:1-7. 10.1016/j.atherosclerosissup.2016.10.002 27998715

[81] Terry SF Krokosky A Rare conditions: where do primary care and genetic diseases intersect? JAAPA. 2010;23:63-4. 10.1097/01720610-201011000-00020 21086896

[82] Timmer MA Blokzijl J Schutgens REG Veenhof C Pisters MF Coordinating physiotherapy care for persons with haemophilia. Haemophilia. 2021;27:1051-61. 10.1111/hae.14404 34492151 PMC9292005

[83] Vandeborne L Van Overbeeke E Dooms M De Beleyr B Huys I Information needs of physicians regarding the diagnosis of rare diseases: A questionnaire-based study in Belgium. Orphanet J Rare Dis. 2019;14:99. 10.1186/s13023-019-1075-8 31054581 PMC6500578

[84] Vieira FS Zucchi P Demandas judiciais e assistência terapêutica no Sistema Único de Saúde. Rev Assoc Med Bras. 2009;55:672-83. 10.1590/S0104-42302009000600011 20191221

[85] Warnick E Umer A Vallejo M Lefeber T Falah N eP439: A survey on the satisfaction of standard primary care residency training in genetics education. Genet Med. 2022;24:S275. 10.1016/j.gim.2022.01.473 PMC9208192 35718772

[86] Wray J Sugarman H Davis L Butler C McIntyre D Hewitt R Improving community-based care for children with a rare condition: The example of long-segment congenital tracheal stenosis and perceptions of health professionals, parents and teachers. Int J Pediatr Otorhinolaryngol. 2021;143:110651. 10.1016/j.ijporl.2021.110651 33662711

[87] Yeung CHT Santesso N Zeraatkar D Wang A Pai M Sholzberg M Integrated multidisciplinary care for the management of chronic conditions in adults: an overview of reviews and an example of using indirect evidence to inform clinical practice recommendations in the field of rare diseases. Haemophilia. 2016;22 Suppl 3:41-50. 10.1111/hae.13010 27348400

[88] Zack P DeVile C Clark C Surtees R Understanding the information needs of general practitioners managing a rare genetic disorder (osteogenesis imperfecta). Community Genet. 2006;9:260-7. 10.1159/000094475 17003536

[89] Ortega Calvo M Gómez-Chaparro Moreno JL González-Meneses López A Guillén Enríquez J Varo Baena A Fernández de la Mota E Mapas conceptuales para el diagnóstico de enfermedades raras en atención primaria. Aten prim (Barc, Ed. impr.). 2012;44:43–50. 10.1016/j.aprim.2011.01.009 PMC7025102 21641688

[90] Carroll JC Hayeems RZ Miller FA Barg CJ Bombard Y Chakraborty P Newborn screening for cystic fibrosis: Role of primary care providers in caring for infants with positive screening results. Can Fam Physician. 2021;67:e144-52. 10.46747/cfp.6706e144 34127476 PMC8202744

[91] García-Ribes M Ejarque I Arenas E Martín V Nuevos retos: el médico de familia ante las enfermedades raras. Aten Primaria. 2006;37:369-70. 10.1157/13087370 16789339 PMC7679924

[92] Rasmussen KW The patient and the relatives could hear the heart beat–a rare case report in primary health care. Lakartidningen. 2009;106:1996. 19764384

[93] Miteva T Jordanova R Iskrov G Stefanov R General knowledge and awareness on rare diseases among general practitioners in Bulgaria. Georgian Med News. 2011;193:16-9. 21617267

[94] Hajek C. Genomic Medicine in Primary Care. S D Med. 2017;62–5. 28817866

[95] Chamsy DJ Kho KA Thoracic endometriosis: A case report. J Minim Invasive Gynecol. 2011;18:S121. 10.1016/j.jmig.2011.08.500

[96] Bermejo E Martínez-Frías ML Prevention, diagnosis and services. Adv Exp Med Biol. 2010;686:55-75. 10.1007/978-90-481-9485-8_4 20824439

[97] Andreasson K Lillpers K Wollheim F Hesselstrand R Systemic sclerosis - a rare but important diagnosis in primary health care. Lakartidningen. 2019;116:FPL6. 31573670

[98] Magrinyá Rull P Altabella Arrufat D. El modelo de atención integral a las enfermedades raras. Inf psiquiátr. 2014;13–20.

[99] Laff M AAFP TAKES LEADERSHIP ROLE WITH LAUNCH OF CENTER FOR DIVERSITY, HEALTH EQUITY. Ann Fam Med. 2017;15:389-90. 10.1370/afm.2032

[100] Kuehne N Hueniken K Xu M Shakik S Vedadi A Pinto D Longitudinal Assessment of Health Utility Scores, Symptoms and Toxicities in Patients with Small Cell Cancer Real World Data. Clin Lung Cancer. 2022;23:e154-64. 10.1016/j.cllc.2021.09.006 34688531

[101] PfizerPfizer Pipeline. 2022. Available: https://cdn.pfizer.com/pfizercom/product-pipeline/Pipeline_Update_28JUL2022_0.pdf?v9aJurw3hqVCO8AwGCx9M_cnB3qJiKCc. Accessed: 18 October 2022.

[102] Emily H. Jung, Alfred Engelberg, Aaron S Kesselheim. Do large pharma companies provide drug development innovation? Our analysis says no. Available: https://www.statnews.com/2019/12/10/large-pharma-companies-provide-little-new-drug-development-innovation/#Reference2. Accessed: 18 October 2022.

[103] Borges do Nascimento IJ Pizarro AB Almeida JM Azzopardi-Muscat N Gonçalves MA Björklund M Infodemics and health misinformation: a systematic review of reviews. Bull World Health Organ. 2022;100:544-61. 10.2471/BLT.21.287654 36062247 PMC9421549

