# Supplementary material for: The utilisation of primary health care system concepts positively impacts the assistance of patients with rare diseases despite limited knowledge and experience by health care professionals: A qualitative synopsis of the evidence including approximately 78 000 individuals
Source: J Glob Health. 2023 Aug 4;13:04030. doi: 10.7189/jogh.13.04030 (PMC10401310; doi:10.7189/jogh.13.04030)
Supplement: Online Supplementary Document [file jogh-13-04030-s001.pdf]

**Supplementary Appendix**

## Table of Contents

***Appendix 1 - Search strategies employed in the scoping review*..................................................*2***

**PubMed = Component 1 AND Component 2 ........................................................................2**

**LILACS/BVS = Component 1 AND Component 2 ..............................................................2**

**Embase = Component 1 AND Component 2 ........................................................................2**

***Appendix 2 - Excluded studies in full-text screening stage with reasons* ...................................*3***

***Appendix 3 – Tentative template of emergency information about patients with rare disease* ...*5***

**Appendix 1 - Search strategies employed in the scoping review**

**PubMed = Component 1 AND Component 2**

Component 1 – Primary healthcare

"Primary Health Care"[Mesh] OR “Care, Primary Health” OR “Health Care, Primary” OR “Primary Healthcare” OR “Healthcare, Primary” OR “Primary Care” OR “Care, Primary” OR "Physicians, Primary Care"[Mesh] OR “Physician, Primary Care” OR “Primary Care Physician*” OR “Primary Care Nursing"[Mesh] OR “Care Nursing, Primary” OR “Nursing, Primary Care”

Component 2 – Rare diseases

"Rare Diseases"[Mesh] OR “Disease, Rare” OR “Rare Disease” OR “Orphan Diseases” OR “Disease, Orphan” OR “Orphan Disease”

**LILACS/BVS = Component 1 AND Component 2**

Component 1 – Primary healthcare

MH: (Primary Health Care) OR (Care, Primary) OR (Care, Primary Health) OR (Health Care, Primary) OR (Healthcare, Primary) OR (Primary Care) OR (Primary Healthcare) OR (Asistencia Primaria) OR (Asistencia Primaria de Salud) OR (Asistencia Sanitaria de Primer Nivel) OR (Atención Básica) OR (Atención Primaria) OR (Atención Sanitaria de Primer Nivel) OR (Primer Nivel de Asistencia Sanitaria) OR (Primer Nivel de Atención) OR (Primer Nivel de Atención de Salud) OR (Primer Nivel de Atención Sanitaria) OR (Primer Nivel de la Asistencia Sanitaria) OR (Atendimento Básico) OR (Atendimento Primário) OR (Atendimento Primário de Saúde) OR (Atenção Básica) OR (Atenção Básica à Saúde) OR (Atenção Básica de Saúde) OR (Atenção Primária) OR (Atenção Primária de Saúde) OR (Atenção Primária em Saúde) OR (Cuidado de Saúde Primário) OR (Cuidado Primário de Saúde) OR (Cuidados de Saúde Primários) OR (Cuidados Primários) OR (Cuidados Primários à Saúde) OR (Cuidados Primários de Saúde) OR (Primeiro Nível de Assistência) OR (Primeiro Nível de Atendimento) OR (Primeiro Nível de Atenção) OR (Primeiro Nível de Atenção à Saúde) OR (Primeiro Nível de Cuidado) OR (Primeiro Nível de Cuidados) OR (Soins de santé primaire) OR MH:N04.590.233.727$ OR MH:SP2.630.121$

Component 2 – Rare diseases

MH: (Rare Diseases) OR (Enfermedades Rara*) OR (Enfermedad Huérfana*) OR (Doenças Raras) OR MH:C23.550.291.906$

**Embase = Component 1 AND Component 2**

Component 1 – Primary healthcare

'primary health care'/exp OR 'first AND line AND care' OR 'health AND care, AND primary' OR 'primary AND care AND nursing' OR 'primary AND healthcare' OR 'primary AND nursing AND care' OR 'general practitioner'/exp OR 'family AND doctor' OR 'family AND physician*' OR 'family AND practitioner*' OR 'general AND physician' OR 'general AND practice AND physician' OR 'general AND practitioners' OR 'physicians, AND family' OR 'physicians, AND primary AND care' OR 'practitioner, AND general' OR 'primary AND care AND doctor' OR 'primary AND care AND physician'

Component 2 – Rare diseases

'rare disease'/exp OR 'disease, rare' OR 'rare diseases' OR 'unusual disease'

## Appendix 2 - Excluded studies in full-text screening stage with reasons

**Supplementary Table 1 – Excluded studies in full-text screening stage with reasons**

| Title | Main author last name | Exclusion reason |
|---|---|---|
| Primary prevention of congenital anomalies: recommendable, feasible and achievable. | Taruscio | 1 |
| Diagnostic evaluation in pulmonary arterial hypertension | Villaquirán-Torres | 2 |
| Primary prevention as an essential factor ensuring sustainability of health systems: the example of congenital anomalies. | Taruscio | 1 |
| Identification of rare diseases using electronic medical records-example of allergic bronchopulmonary aspergillosis in UK primary care data | Maguire | 1 |
| Late referral of lupus to rheumatologists in India: A study of lupus cohort | Chandrashekara | 1 |
| Exploring the cost and burden of illness of hereditary angioedema in England | Helbert | 1,2 |
| Improving newborn screening follow-up in pediatric practices: Quality improvement innovation network | Hinton | 1 |
| Red flags for the differential diagnosis of granulomatous mastitis: a case report. | Chalmers | 1 |
| COVID-19 and children with congenital anomalies: a European survey of parents' experiences of healthcare services | Latos-Bieleńska | 1 |
| Microscopic polyangiitis: Report of a young woman presenting initially with severe localized myalgia | Tamura | 1 |
| Assistência à pessoa com condições crônicas na Atenção Primária à Saúde | Lieberenz | 2 |
| How genomic information is accessed in clinical practice: an electronic survey of UK general practitioners | Evans | 1 |
| European recommendations for primary prevention of congenital anomalies: a joined effort of EUROCAT and EUROPLAN projects to facilitate inclusion of this topic in the National Rare Disease Plans. | Taruscio | 1 |
| Health economic benefits through the use of diagnostic support systems and expert knowledge. | Willmen | 1,2 |
| Integrating medical and genomic data: a successful example for rare diseases. | Dias | 1 |
| Shared communication processes within healthcare teams for rare diseases and their influence on healthcare professionals' innovative behavior and patient satisfaction. | Hannemann-Weber | 1 |
| Cross-border health care represents a key issue in the field of rare diseases | Facchin | 11 |
| How are patients with rare diseases and their carers in the UK impacted by the way care is coordinated? An exploratory qualitative interview study. | Simpson | 1 |
| Proposta de seminários para formação de teleconsultores sobre a Política Nacional de Atenção Integral às Pessoas com Doenças Raras no SUS | Programa Nacional de Telessaúde Brasil Redes | 1 |
| Prevalence of zebras in general practice: Data from the Continuous Morbidity Registration Nijmegen | Van de Laar | 2 |
| New Tools and Approaches for Family Physicians. | Seehusen | |
| Falling short: The NHS workforce challenge: Workforce profile and trends of the NHS in England | Buchan | 1,2 |
| People's Republic of China health system review | Qingyue | 2 |
| The role of unregistered healthcare workers in preventing readmission to hospital: A systematic review protocol | Moss | 2 |
| Can the use of urgent care clinics improve access to care without undermining continuity in primary care? | Villasenor | 2 |
| Nursing challenges for universal health coverage: A systematic review | Schveitzer | 1,2 |
| Approaches to improving the contribution of the nursing and midwifery workforce to increasing universal access to primary health care for vulnerable populations: A systematic review | Dawson | 2 |
| Do patient-centered medical home access and care coordination measures reflect the contribution of all team members? A systematic review | Annis | 2 |
| Systematic review of interventions to increase the delivery of preventive care by primary care nurses and allied health clinicians | McElwaine | 2 |
| Nursing interventions in monitoring the adolescent with Cystic Fibrosis: A literature review | Reisinho | 1 |
| The effect of pharmacist-led interventions in optimising prescribing in older adults in primary care: A systematic review | Riordan | 2 |
| Interventions for compassionate nursing care: A systematic review | Blomberg | 1,2 |
| The impact of transitional care programs on health services utilization in community-dwelling older adults: A systematic review | Weeks | 1 |
| Nurses as substitutes for doctors in primary care | Laurant | 2 |
| Falling short: The NHS workforce challenge: Workforce profile and trends of the NHS in England | Buchan | 1,2 |
| Nursing interventions in monitoring the adolescent with Cystic Fibrosis: A literature review | Reisinho | 1 |
| Palliative care interventions for people with multiple sclerosis | Latorraca | 1 |

| | | |
|---|---|---|
| Information provision for people with multiple sclerosis | Kopke | 1 |
| Health information technology continues to show positive effect on medical outcomes: Systematic review | Kruse | 1,2 |
| Indicators of good nursing practices for vulnerable groups in primary health care: A scoping review | Egry | 2 |

Legend:
1 Not primary healthcare
2 Not rare diseases

## Appendix 3 – Tentative template of emergency information about patients with rare disease

**Identification** *(Identificacion, Identificação)*

**Name** *(Nombre, Nome)* **Age** *(Edad, Idade)*

**Emergency contact** *(Contacto de emergencia, Contato de emergência)*

**Address** *(Dirección, Endereço)*

**Main rare disease** *(Enfermedad rara principal, Doença rara principal)*

**Brief pathophysiology of the disease** *(Patofisiología de la enfermedad, patofisiologia da doença)*

**Current treatment** *(Tratamiento actual, Tratamento atual)*

**Primary healthcare providers** *(Profesional de salud responsable, Profissional de saúde responsável)*

**Physician 1** *(Medico, medico)*__________ *Phone (Teléfono, telefone)*__________

**Physician 2** *(Medico, medico)*__________ *Phone (Teléfono, telefone)*__________

**Physician 3** *(Medico, medico)*__________ *Phone (Teléfono, telefone)*__________

**Other** *(Otro, outro)* *Phone (Teléfono, telefone)*

**Alergies** *(Alergias, alergias)*

**Previous emergency issues** *(Situaciones de emergencias pasadas, eventos de emergência prévios)*

**1.**
**2.**
**3.**
**4.**
**5.**

**List of potentially threatening acute emergencies based on the literature**
*(Lista de emergencias potencialmente severas dichas en la literatura)*
*(Lista de emergências potencialmente graves previstas na literatura)*

**Medications NOT INDICATED in ANY emergency**
*(Medicamentos CONTRA INDICADOS in cualquiera situación de emergencia)*
*(Drogas CONTRA-INDICADAS EM QUALQUER situação de emergencia)*

**More information can be access on** *(Más contenido están disponibles en, mais informações podem ser acessadas em)*

**Orphanet:** https://www.orpha.net/consor/cgi-bin/index.php **and** https://www.orpha.net/consor/cgi-bin/Disease_Emergency.php?lng=EN

**FEDER:** https://www.enfermedades-raras.org

**NORD:** https://rarediseases.org/for-patients-and-families/information-resources/rare-disease-information/

**Primarily designed by Lemos et al. 2022**
